# Dermoscopy of Generalized Eruptive Histiocytosis: Case Report and Brief Review of the Literature

**DOI:** 10.5826/dpc.1003a57

**Published:** 2020-06-29

**Authors:** Danijela Dobrosavljevic, Jovana Majstorovic, Martina Bosic

**Affiliations:** 1 Clinic of Dermatovenereology, Clinical Centre of Serbia, Belgrade, Serbia; 2 Faculty of Medicine, University of Belgrade, Serbia; 3 Institute of Pathology, University of Belgrade, Serbia

**Keywords:** generalized eruptive histiocytosis, non-Langerhans cell histiocytosis, histiocytic disorders, dermoscopy, dermatoscopy

## Introduction

Generalized eruptive histiocytosis (histiocytoma) (GEH) is a very rare cutaneous non-Langerhans cell histiocytosis, characterized by recurrent crops of small red to brown papules.

## Case Presentation

**Figure 1:**
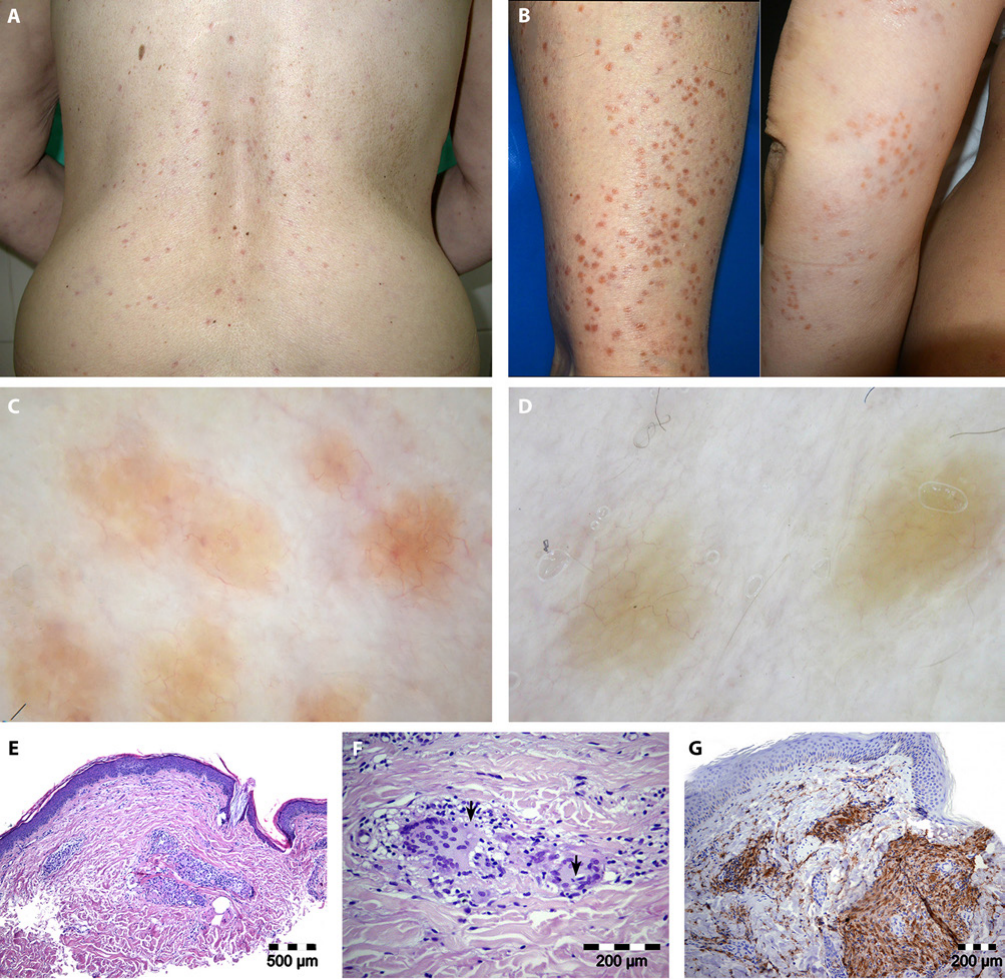
(A,B) Symmetric yellow-brown, flat-topped papules localized on the trunk (A) and extremities (B). (C) Dermoscopic finding: orange-yellow homogeneous pigmentation, delicate linear branching, and serpentine vessels. Solitary, red clods are present in some lesions. (D) Dermoscopic finding after 3 months: partially regressed lesions reveal yellow background and linear serpentine vessels. (E) Focally dispersed small dermal granulomas composed of histiocytes and multinuclear giant cells with peripheral arrangement of nuclei (E,F) (H&E, ×100). (F) Focal emperipolesis is noted in giant cells (arrows) (H&E, ×400). (G) Positive immunohistochemical staining of CD68 in granulomas (magnification ×200). Immunohistochemically, granulomas presented with profile CD68+ CD163+/−, and CD1a−.

A 58-year-old woman with an 8-month history of hundreds of symmetric yellow-brown flat-topped papules (Figure 1, A and B) came for dermatologist consultation. The lesions appeared in crops localized on the trunk and extremities. It was believed that the skin lesions were caused by trazodone and lithium, which were being used in the treatment of bipolar disorder. The medications were stopped, but the lesions continued to develop. Routine blood and urine analyses were unremarkable.

Dermoscopy was performed on one representative lesion on the lower leg. The histology was consistent with the diagnosis of GEH (Figure 1, C–G).

After GEH diagnostics, additional investigations were done. Hyperprolactinemia, hypercorticism, and hypofunction of the thyroid gland were detected. Abdominal ultrasonography and MRI of the sella turcica and hematological studies with biopsy of the bone marrow were without pathological findings. Three years from the first onset, the skin lesions mostly resolved, leaving hyperpigmented macules.

## Literature Review

Seventy-four cases (58.1% males) of GEH have been published, including 24 (32.4%) children (Table 1 and Supplementary References, which are appended to the pdf). The average age was 30.5 years. In adults and children the average age was 43.2 years and 4.2 years, respectively. The most frequent body site was the trunk (86%), followed by the extremities (79%). The lesions resolved spontaneously from 2 weeks onward, but in rare cases persisted for 20 years. In 4 children the lesions evolved into xanthoma disseminatum; in 1 child the lesions coexisted with juvenile xanthogranuloma.

Although GEH is a benign, self-healing eruption of non-Langerhans cell lineage, follow-up is necessary.

Two age groups of GEH patients are reported: up to 14 years and adults. Brain infiltrations and diabetes insipidus are reported in 3 (12.5%) children up to age 4 years with xanthomatous evolvement. In 5 (10.0%) of the published adult cases, hematological disorders of myeloid lineage such as acute monocytic/monoblastic leukemia (2 cases) or chronic myelomonocytic/eosinophilic leukemia (3 cases) are reported (Table 1). In preschool children, diabetes insipidus should be suspected if GEH evolves into xanthoma. In adults, hematological follow-up is suggested.

Clinical differential diagnosis of GEH includes other histiocytic disorders such as Letterer-Siwe disease, juvenile xanthogranuloma (multiple), papular xanthoma, and progressive nodular histiocytosis. Exanthema due to medications and viruses, with separate entity Gianotti-Crosti syndrome and early eruption of guttate psoriasis, are main differentials as well.

A case describing dermoscopy of GEH lesions resembling molluscum contagiosum in an infant has been published [1]. A homogeneous orange-yellow pattern with an erythematous border described as “setting-sun” was recognized. Histology revealed histiocytic cells with foamy xanthomatous cytoplasm [1]. The dermoscopic finding in our case presented with orange-yellow homogeneous pigmentation, delicate linear branching, serpentine vessels, and solitary, red clods. Histology revealed histiocytic cells forming granulomas.

The dermoscopic differential diagnosis of GEH is broad and encompasses juvenile xanthogranuloma, cutaneous sarcoidosis, necrobiosis lipoidica, granuloma annulare (palisading granuloma histological subtype), elastosis perforans serpiginosa, granulomatous rosacea, annular elastolytic giant cell granuloma, and rheumatoid nodules. Among infective diseases, lupus vulgaris, cutaneous leishmaniasis, borderline tuberculoid leprosy, and Majocchi granuloma are the most important differentials [2]. Combining clinical, dermoscopic, and histological findings is of greatest importance in any of the diseases mentioned.

## Conclusions

Our case emphasizes the importance of dermoscopic examination in the everyday practice of dermatologists. Further studies of skin histiocytic disorders are required in order to establish all dermoscopic criteria.

## Figures and Tables

**Table 1:** Clinical Characteristics of the Published Cases With Generalized Eruptive Histiocytosis (Histiocytoma) (GEH)

Reference^a^	Age at Onset (yrs)^b^	Sex	Location	Course	Associated Finding
Wise (1919)^c^	22	M	Trunk, proximal part of extremities	Lasted 20 years	None
Glauberzon and Lebedeff (1952)^c^	34	F	Disseminated	Not defined	None
Calas et al (1959)^c^	52	M	Face, trunk, extremities	Not defined	None
Baccaredda-Boy (1960)^c^	33	M	Trunk, extremities, face, mouth	No data, died after 19 years	Mutilating polyarthritis
Herzberg (1961)^c^	30	F	Trunk, extremities	Spontaneous regression after 13 years	Amenorrhea
Winkelmann and Muller (1963)	51	F	Trunk, extremities	Number of lesions increased within 15 months	Osteoarthritis
Winkelmann and Muller (1963)	58	M	Trunk, axillae, pubic area and penis, buccal mucosa	Increasing in number within 4 years	None
Winkelmann and Muller (1963)	38	F	Face, trunk, extremities	Cleared gradually after 12 years	None
Cramer (1963)	25	M	Disseminated	Resistant to steroids	None
Pegum (1973)	42	M	Trunk, extremities	No regression after 2.5 years	High cholesterol
Sohi et al (1979)	49	M	Generalized	Regressed in a few months, then recurred 2 years later	None
Winkelmann (1980)	3 months	F	Extensor limbs, buttocks	Persisted until at least 9 years old	Glaucoma and uveitis
Caputo et al (1981)	25	M	Thorax, abdomen, inguinal fold, proximal extremities	Resolved spontaneously in 4 years	None
Aso et al (1982)	4	M	Disseminated	Spontaneous regression of 80% lesions after 2 years	None
Arnold et al (1982)	32	M	Disseminated	Persisted for 20 years	None
Bobin et al (1983)	22	M	Trunk, extremities	Partial regression within 1 year	None
Idikio and Hogan (1983)	57	F	Abdomen, pubic area, breasts, back, axillae, face	Unchanged in 9 years	Hyperlipidemia type IV
Statham et al (1984)	66	F	Trunk, extremities, nasal mucosa	Death 18 months after the diagnosis from acute leukemia	Acute leukemia of monoblastic/histiocytic origin
Caputo et al (1987)	11 months	M	Trunk, neck, head, extremities	Disappeared in 5 years	None
Caputo et al (1987)	11 months	F	Face, trunk	Mostly regressed in 6 years	None
Caputo et al (1987)	10 months	M	Trunk, scrotum	Mostly regressed in 2 years	None
Caputo et al (1987)	44 months	F	Trunk, axillae, face	Mostly regressed in 3 years	None
Shimizu et al (1987)	24	M	Cheeks, later generalized	Regressed in 1 year	None
Sigal-Nahum et al (1987)	7	F	Face, buttocks, extremities	Spontaneous regression and new crops within few months	None
Braun-Falco et al (1988)	19	M	Trunk, axillary, face, throat	Evolved into xanthoma disseminatum	None
Grob et al (1988)	25	M	Face, axillary	Eruption persisted 2 years	None
Umbert and Winkelmann (1989)	67	F	Face, trunk, arms	Slow progression within 9 years	Hypothyroidism, normolipemic xanthelasma, polyclonal gammopathy
Ashworth et al (1990)	21	M	Disseminated, sparing mucosa	8 years continual progression of the disease	Atopic dermatitis, asthma
Saijo et al (1991)	14	F	Trunk	Within 14 months: partial regression with new lesions	None
Stables and MacKie (1992)	55	F	Arms, trunk, upper thighs, face	Present for at least 24 months	None
Izaki et al (1993)	1	M	Face, neck, upper arms	Persisted for next 5 years until spontaneous resolution	None
Goerdt et al (1994)	69	M	Trunk, extremities	Persisted for at least 5 years	High cholesterol
Repiso et al (1995)	4	M	Face, trunk, proximal extremities	Evolved into xanthoma disseminatum	Developed diabetes insipidus and brain infiltrations
Gibbs and O’Grady (1996)	41	M	Face, arms, torso	Lesions persisted for 9 years	Diabetes mellitus type II
Jang et al (1999)	3 months	M	Face, trunk, groin, upper and lower limbs	Spontaneous regression within 2 months; no new lesions within 2 years	None
Matsushima et al (1999)	5	F	Generalized	Spontaneous regression within 8 months	Rheumatic fever
Wee et al (2000)	9	M	Trunk, proximal extremities	Spontaneous regression of some lesions with new crops	None
Marzano et al (2002)	33	F	Trunk, extremities	Resolved within 6 months	None
Wollenberg et al (2002)	13	F	Abdomen, trunk	Persisted for 3 years	Coexistence with skin lesions proven to be indeterminate cell histiocytosis
Klemke et al (2003)	59	M	Trunk, neck, face, and thighs	Improved under aplasiogenic regimen	Acute monocytic leukemia
Seward et al (2004)	55	M	Trunk, extremities	Several lesions resolved after cryotherapy	None
Deng et al (2004)	39	M	Face, trunk, limbs	Spontaneously resolved within 6 months	Increased eosinophilia
Mehravaran (2004)	53	F	Trunk, upper extremities	No follow-up	None
Tamiya et al (2005)	14 months	F	Trunk, extremities	Spontaneously resolved within 1 month	Immunoglobulin G, human herpesvirus 6
Vázquez-Blanco et al (2006)	64	M	Trunk, extremities, mucous membranes	Subsided after photochemotherapy, but reappeared	None
Kiliç et al (2006)	1	M	Face, trunk, extremities	Stable for 12 months, partial regression after 41 months	None
Lan Ma et al (2007)	32	F	Trunk, extremities, face	In 3 months resolved with PUVA	Eosinophilia in peripheral blood and in bone marrow cytology
Tang et al (2007)	36	F	Trunk, abdomen, extremities	After 8 years, spontaneous regression of some lesions observed	None
Fernández-Jorge et al (2007)	41	F	Trunk and arms	Spontaneously resolved in 11 months	Hypercholesterolemia
Bajaj and Iqbal (2008)	28	M	Face, chest, axillae	Resolved after 1 week with liquid nitrogen	None
Kwinter and DeKoven (2009)	53	F	Face, neck, arms	Resolved after 8 months with isotretinoin, then recurred	None
Chern et al (2010)	5 months	F	Face, trunk, arms, legs	Spontaneously resolved in 6 months	Mild leukocytosis
Aggarwal et al (2010)	61	M	Trunk, arms, legs	Spontaneously resolved with relapses within 4 years	None
Sharath Kumar et al (2011)	23	F	Face, trunk, arms, legs	Minimal resolution, persisted 5 years	None
Attia et al (2011)	48	F	Upper limbs and trunk	Spontaneously resolved	None
Montero et al (2012)	80	M	Trunk, abdomen	Resolved after 6 months	Chronic myelomonocytic leukemia
Verma (2012)	10	M	Hands, feet, trunk	Coexistence with juvenile xanthogranuloma lesions	None
Cardoso et al (2013)	79	M	Trunk, eyelids	Spontaneously resolved in 2 months	None
Zamudio Vega et al (2013)	8	M	Face, upper extremities	8 months unchanged	None
Shon et al (2013)	84	M	Face, neck, arms	Died after 4 months	Chronic myelomonocytic leukemia
Kazi et al (2014)	23	M	Lower extremities	No follow-up	None
Ghandi et al (2015)	28	F	Face, trunk, extremities	Spreading within 2 years	None
Ziegler et al (2015)	20	M	Trunk, extremities	Complete remission with imatinib	*FIP1L1-PDGFRA-*positive chronic eosinophilic leukemia
Hansel et al (2015)	60	M	Trunk, extremities	Remission with PUVA and topical corticosteroids	None
Mahajan et al (2015)	60	M	Extremities	No follow-up	None
Wilk et al (2016)	64	M	Trunk, extremities	Unchanged for several years	None
Piney et al (2016)	28	M	Face, trunk, extremities	Resistant to imatinib, interferon alpha, anakinra; resolved after PUVA therapy	Arthralgia
Alperovich et al (2017)	3	F	Trunk, face	6-month follow-up: CNS lesions-xanthomata	Diabetes insipidus
Arif et al (2017)	26	F	Face, trunk, arms	Persisted and coalesced within 3 months	None
Kar et al (2018)	6	F	Face, axillae, trunk	Spontaneously resolved	None
Kaçar et al (2018)	19 months	F	Diaper area, extremities, trunk	Spontaneously resolved	None
Costin et al (2019)	24	F	Trunk, neck and proximal upper extremities	Unchanged after 1 year	None
Takahashi et al (2019)	1	M	Trunk	After 1 year partially regressed	None
Kobayashi et al (2019)	7 months	M	Neck, trunk, extremities	2-year follow-up; lesion evolved into xanthoma disseminatum; resistance to chemotherapy	Diabetes insipidus

a Full references are provided in the supplementary content, which is appended to the pdf.

b Age is given in years except where indicated as months.

c In 1963 Winkelmann and Muller reported 3 cases of GEH, but some cases with cutaneous and histological abnormalities consistent with GEH were reported before their report, under different names (Sohi et al, *Dermatologica*. 1979;159(6):71–75; Bobin et al, *Ann Dermatol Venereol.* 1983;110(10):817–824).
